# Correlation between Topographic Vessel Density and Retinal Thickness Changes in Patients with Diabetic Macular Edema Treated with Anti-VEGF Therapy: Is It a Suitable OCTA Biomarker?

**DOI:** 10.3390/jpm13121718

**Published:** 2023-12-16

**Authors:** Juan Santamaría, José María Caminal, Estefanía Cobos, Marc Biarnes, Ramon Rodriguez-Leor, Rahul Morwani, Manel García-Mendieta, Daniel Lorenzo, Pere García-Bru, Luis Arias

**Affiliations:** 1 Department of Ophthalmology, Ocular Oncology and Vitreoretinal Service, Bellvitge University Hospital, L’Hospitalet de Llobregat, 08907 Barcelona, Spain; jsantamariaa@bellvitgehospital.cat (J.S.); ecobos@bellvitgehospital.cat (E.C.); rmorwani@bellvitgehospital.cat (R.M.); dlorenzo@bellvitgehospital.cat (D.L.); p.garcia@bellvitgehospitcal.cat (P.G.-B.); luisariasbarquet@gmail.com (L.A.); 2 OMIQ Research, c/Tamarit 39, 08205 Sabadell, Spain; mbiarnes@omiq.es; 3 Oslo Universitetssykehus Rikshospitalet, Ophthalmology, 0372 Oslo, Norway; ramond@ous-hf.no; 4 Hospital de Sant Pablo y Santa Tecla, Ophthalmology, 43003 Tarragona, Spain; mgarciamen@xarxatecla.cat

**Keywords:** diabetic macular edema, vessel density, optical coherence tomography angiography, anti-VEGF inhibitors

## Abstract

The objective of this study was to determine the correlation between topographic vessel density (VD) and retinal thickness (RT) reductions induced by vascular endothelial growth factor inhibitors (anti-VEGF) in patients with diabetic macular edema (DME) using optical coherence tomography angiography (OCTA). This was a prospective, interventional case series. VD and RT measurements were separately taken in four parafoveal subfields at baseline and after six months of treatment. This correlation was statistically assessed using Spearman’s rho correlation coefficient after adjustment for multiple comparisons. The study included a total of 48 eyes in the final analysis. Mean VD decreased from baseline to month 6 (from 45.2 (±3.5) to 44.6% (±3.2) in the superficial capillary plexus and from 50 (±3.3) to 49% (±3.9) in the deep capillary plexus). Statistically significant reductions in RT were observed in all ETDRS sectors (*p* < 0.0001). No significant association was found between RT and VD, even when analyzing responders and non-responders separately. After six months of anti-VEGF treatment, no significant correlation was observed between the topographic VD and RT values. These findings suggest that reductions in VD values may not solely result from a reduction in microaneurysms, also being affected by the repositioning of displaced vessels due to edema and a reduction in their caliber. Therefore, VD changes may not be a suitable indirect OCTA biomarker of microaneurysm turnover and treatment response.

## 1. Introduction

Diabetic macular edema (DME) is a major complication in patients with diabetic retinopathy (DR) that can lead to vision loss. DME occurs due to a disruption of the blood retinal barrier in areas with vascular anomalies such as telangiectatic vessels and microaneurysms (MAs). These alterations are directly associated with disease severity and progression [1].

Currently, the gold standard technique for identifying leaking vessels and ischemia (macular and peripheral) is fluorescein angiography (FA). However, this imaging technique has several important drawbacks: it is time-consuming, invasive, and may cause complications [2]. With the introduction of optical coherence tomography angiography (OCTA), a non-invasive tool has become available to visualize both the superficial and deep capillary plexus separately, which is not possible with FA.

Despite the potential of evaluating different OCTA biomarkers and the possibility to compare follow-up images, there are currently no OCTA guidelines that allow for disease activity and response to treatment to be assessed properly. In contrast, structural optical coherence tomography (OCT) has well-defined features that can be measured during follow-up, such as retinal thickness (RT), subfoveal neuroretinal detachment, and intraretinal cysts, among others [3,4].

In OCTA, vessel density (VD) is defined as the proportion of the vessel area showing blood flow relative to the total area measured [5,6,7]. Several studies have found that eyes with DR and DME have lower VD than controls and that this characteristic is directly associated with the risk of retinopathy progression and the likelihood that treatment will be needed in the near term [8,9].

**Figure 1:**
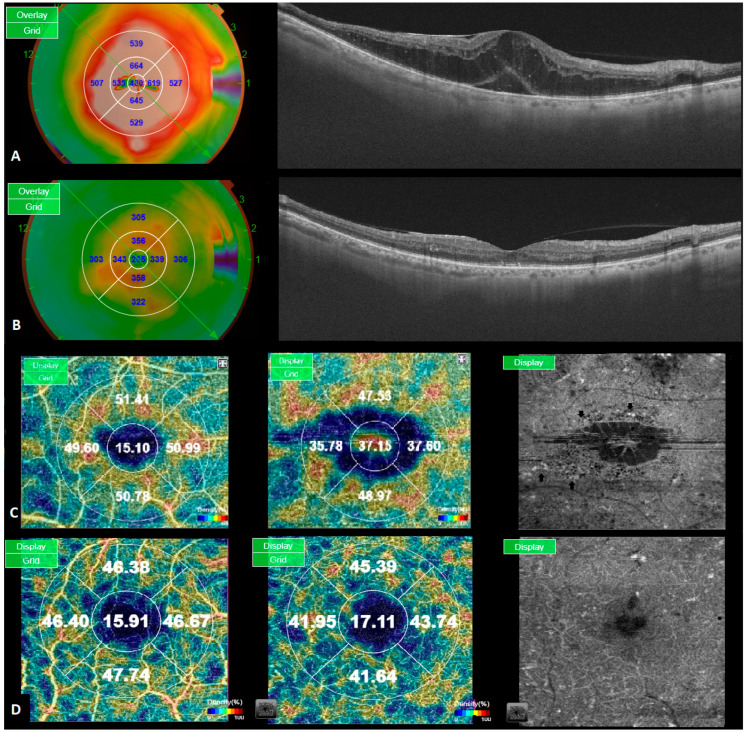
(**A**): Pre-treatment ETDRS retinal thickness grid with the corresponding B-scan. (**B**): Post-treatment ETDRS retinal thickness grid with the corresponding B-scan. (**C**): Pre-treatment optic coherence tomography angiography (OCTA) vessel density (VD) map of the superficial (SCP) and deep capillary plexus (DCP), along with en face OCTA of the DCP. (**D**): Post-treatment optic coherence tomography angiography (OCTA) vessel density (VD) map of the superficial (SCP) and deep capillary plexus (DCP), along with en face OCTA of the DCP. After six months of treatment, there is a reduction in VD values in all subfields at the SCP level, whereas the DCP exhibits asymmetric changes, primarily due to the repositioning of centrally displaced vessels. Additionally, there is a reduction in the number of microaneurysms located near cystic spaces, as shown in the en face OCTA.

DME is not a homogenous disease; areas of capillary dropout and ischemia coexist with areas of vascular tortuosity and MAs. Furthermore, the presence of edema modifies the normal macular vascularization in an asymmetrical manner. As a result, different vessel density (VD) patterns with areas of abnormally high and low VD values can be seen in the same eye (Figure 1).

Previous studies have shown that areas with increased number of MAs and dilated shunt vessels, which are prone to leakage, are localized in the border areas of the DME, that their number can be modified with treatment (MAs turnover), and that areas with residual edema after anti-vascular endothelial growth factor (VEGF) therapy are characterized by a higher density of MAs [10,11].

The aim of this study was to investigate whether areas of increased VD values, presumably due to the presence of a higher density of MAs, as well as displaced and telangiectatic vessels, are topographically correlated with areas of increased RT and whether the changes in VD values after treatment are correlated with a better response to treatment in terms of RT reduction.

## 2. Materials and Methods

This prospective, interventional study was conducted at the Bellvitge University Hospital (Barcelona, Spain) from May 2018 to November 2020. All patients signed an informed consent form before enrollment. All patient-related data were anonymized for this analysis. The research protocol adhered to the principles of the Declaration of Helsinki and was approved by the Clinical Research Ethics Committee at Bellvitge University Hospital.

### 2.1. Study Population

The following data were collected at baseline: age; sex; type of diabetes; glycated hemoglobin (HbA1c) levels; lens status; and stage of retinopathy, which was recorded as either non-proliferative diabetic retinopathy (NPDR; mild, moderate, severe) or proliferative DR (PDR). Best-corrected visual acuity (BCVA) was determined according to the standard Early Treatment Diabetic Retinopathy Study (ETDRS) protocol. Intraocular pressure (IOP) was also assessed. Color fundus photography, OCT imaging, and OCTA imaging were performed after pharmacological mydriasis.

The inclusion criteria were as follows: age ≥ 18 years; treatment-naïve; and diagnosis of center-involving DME (defined as retinal thickening, intraretinal cysts, hyperreflective foci, or subretinal fluid documented by OCT, regardless of DR severity). The exclusion criteria were as follows: best-corrected visual acuity (BCVA) < 35 ETDRS; vitreomacular traction or tractional retinal detachment on OCT; choroidal neovascularization or any other retinal vascular disease; poor-quality OCTA images (signal strength intensity [SSI] < 40 due to media opacity or significant motion artifacts); previous history of any macular treatment (e.g., intravitreal anti-VEGF, corticosteroids, laser therapy, or vitrectomy).

All patients received three consecutive monthly injections of an anti-VEGF agent (ranibizumab or aflibercept) and continued with a bimonthly treatment regimen. BCVA and OCTA were performed at baseline and after 6 months of treatment. A normative database of macular VD values in healthy subjects for OCT Triton [12] was used to establish the mean normal value for each quadrant. Pre- and post- treatment VD were obtained to assess the deviation from normal values at baseline and post-treatment changes.

### 2.2. Image Acquisition

The swept-source (SS) OCT Triton (Topcon Corporation, Tokyo, Japan) was used to perform the SS-OCT and SS-OCTA measurements. This device uses a central wavelength of 1050 nm with an axial resolution of 8 μm, a transverse resolution of 20 μm, and a scanning speed of 100,000 A-scans per second. The OCTARA image processing algorithm was used. The SMARTTrack™ eye tracking technology was used to ensure that all follow-up OCTA exams were performed in the same position as in previous exams. A 6 mm × 6 mm scan in “angio-macula” mode was automatically centered at the fovea.

### 2.3. OCT Parameters

The following pre- and post-treatment parameters were recorded: central subfield retinal thickness (CST) measured within 1 central mm and four parafoveal subfields (superior, inferior, nasal, and temporal) using an ETDRS grid overlay comprising the two inner rings.

### 2.4. OCTA Parameters

Automated segmentation was used to evaluate the SCP and DCP. Segmentation errors were corrected manually. The SCP was segmented from 2.6 μm beneath the internal limiting membrane to 15.6 μm beneath the interface of the inner plexiform layer (IPL) and inner nuclear layer (INL). The DCP was segmented from 15.6 μm beneath the IPL/INL to 70.2 μm beneath the IPL/INL. VD measurements of the SCP and DCP were calculated automatically using the built-in software (OCTA-ratio analysis). VD measurements were made separately in four parafoveal subfields (superior, inferior, nasal, and temporal) using an ETDRS grid overlay comprising the two inner rings.

### 2.5. Statistical Analysis

Descriptive statistics were used to characterize the sample using the mean (standard deviation [SD]) or median (interquartile range [IQR]) for quantitative variables, depending on their distribution, and n (percentage) for categorical variables. The comparison between baseline and final (after DME treatment) BCVA and retinal thickness in each subfield (central, superior, inferior, nasal, and temporal) was conducted using Wilcoxon tests after confirming that these did not have a normal distribution using Shapiro–Wilk tests.

The association between retinal thickness (RT), as measured via SD OCT, and VD, determined using OCTA imaging in each of the four sectors (superior, inferior, nasal and temporal) of the ETDRS chart and in the SCP and the DCP, was statistically assessed using Spearman’s rho correlation coefficient after adjustment for multiple comparisons using the Bonferroni method. For statistically significant results, scatterplots between RT and VD with LOWESS regression lines were plotted. This association was assessed in three scenarios: (1) at month 0 or pre-DME treatment; (2) at month 6 after DME treatment; and (3) for change in retinal thickness and in VD (month 6–month 0) after DME treatment.

To determine if the change in VD differed by treatment response, we first defined responders as those with a CST decrease > 50 μm after therapy and non-responders as all others [13]. Then, we used mixed-effects models (which account for the correlation between eyes of a given individual in bilateral cases [14], considering the patient level as a random effect and using maximum likelihood estimation methods) with VD change (VD at month 6–VD at month 0) as the outcome of interest and treatment response (as defined previously) as the independent variable. These analyses were conducted separately for each sector and for both plexuses: SCP and DCP.

We estimated the required sample size of the study. The assumptions included an α = 0.05, a β = 0.20 (for a power of 80%), a difference between pre/post periods of 2% with a standard deviation (SD) of 5%, and a discontinuation rate of 10%, with a two-tailed contrast. These assumptions led to the recruitment of at least 55 eyes for the study. The GRANMO software (version 7.12) was used for these calculations. A total of 43 patients (63 eyes) were initially enrolled in the study. A comprehensive medical history and ophthalmological evaluation were performed in all cases.

The analyses were conducted using Stata IC, version 15.1 (StataCorp; College Station, TX, USA). A two-tailed *p*-value < 0.05 was considered statistically significant.

## 3. Results

The sample included 48 eyes from 34 patients, 29.4% of whom (10/34) were females and whose mean age was 63.7 years (SD 10.5). In the sample, 94.1% of patients (32/34) had type 2 diabetes mellitus, and their mean HbA1C was 7.84% (SD 2.08%), while their eyes had a mean BCVA of 69.7 letters (SD 11.2) and a mean central RT of 376.8 μm (SD 100.3). Further details are provided in Table 1.

At baseline, the median BCVA was 71.5 letters (IQR 14), and this decreased at six months to 66.5 letters (IQR 16), although this difference was not statistically significant (*p*-value = 0.12). Table 2 shows the changes in VD between baseline and month 6 by location and plexus (SCP and DCP).

VD generally decreased from baseline to month 6, but statistically significant differences were observed only for the nasal sector, with a 2.9% decrease (*p* = 0.001) and a small, borderline significant decrease in overall mean VD in the DCP of 1.1% (*p* = 0.055) (Table 2). The median CST decreased from 354.5 μm (IQR 82.5) at baseline to 306 μm (IQR 86) at six months (*p*-value < 0.0001). Decreases in RT in all other ETDRS sectors were statistically significant (*p* < 0.0001; Table 3).

The association between RT on SD OCT and VD on OCTA is shown in Table 4.

After Bonferroni adjustment, a statistically significant and moderate association was shown only for the inferior sector in the SCP at baseline (rho = 0.43, *p*-value = 0.002). No other statistically significant association was found in any particular location.

Based on changes in CST due to anti-VEGF therapy at month 6, 21 eyes (21/48, 43.8%) were considered responders, and 56.3% of this study’s subjects were considered non-responders. Table 5 shows the results of the differences between the responders and non-responders in the mixed-effects models for each location and in the SCP and the DCP. No statistically significant differences between these two groups were found (all *p*-value > 0.05), suggesting that VD change did not differ between the responders and non-responders in any sector or plexus after 6 months of anti-VEGF therapy in patients with DME.

DR results from the changes in retinal microvascular structures due to persistent hyperglycemia. Ultimately, this leads to the presence of capillary dropout and retinal ischemia, the appearance of vascular anomalies such as vascular tortuosity, intraretinal microvascular anomalies (IRMAS), and MAs which are prone to leakage. DME is caused by increased concentrations of VEGF, which induces an increase in permeability, mainly from Mas and compromised vessels due to the disruption of the blood–retinal barrier [15].

Given that the density of MAs is higher in edematous areas and that their reduction after treatment is directly related to the degree of RT reduction, in this investigation, we sought to determine if there is a relation between sectorial VD changes and the degree of RT reduction after treatment. There were statistically significant decreases in RT in all ETDRS sectors after six months of treatment. Regarding the relation between RT and VD, no statistically significant association was found in any particular location, with the exception of the inferior sector in the SCP at baseline (rho = 0.43; *p*-value = 0.002). We did not find differences between responders and non-responders in terms of VD reduction after treatment.

### 3.1. Location of MAs and Their Influence in DME

OCTA studies have demonstrated that approximately 80% of MAs are located at the level of the DCP, which is the most significantly impaired retinal layer mainly due to its structure, which is predominantly composed of capillaries surrounded by pericytes that are prone to damage due to hyperglycemia [16,17,18,19]. The increased number of MAs in the DCP is associated with macular volume; thus, the MAs in the DCP contribute to the pathogenesis of DME, especially the cystoid type [19].

**Figure 2:**
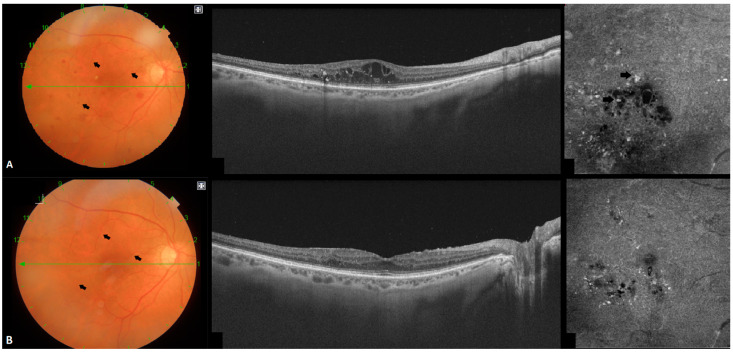
(**A**): Pre-treatment fundus photography with its corresponding B-scan and en face optic coherence tomography angiography (OCTA). Arrows indicate microaneurysms (MAs) and tortuous vessels in fundus photography. MAs are also shown adjacent to cystic areas in the en face OCTA. (**B**): Post-treatment fundus photography with a corresponding B-scan and en face OCTA. Notice the turnover of MAs and the reduction in vessel tortuosity in fundus photography. There is also a reduction in MAs, especially those located near cystic areas in the en face OCTA.

MAs are often detected at the borders of the edema; this higher peripheral concentration and the presence of active leakage is what contributes to the expansion of edematous areas [20]. During DME evolution, the appearance and disappearance of MAs (MAs turnover) is a dynamic process (Figure 2). Their increase reflects DR and DME progression [21,22], whereas a reduction after treatment may be a potential treatment response biomarker [23,24].

During anti-VEGF therapy, RT reduction is not symmetric among all regions, with the less responsive areas being the ones with a higher density of MAs, whereas responsive areas tend to have a lower density of MAs [25].

### 3.2. VD and the Risk of Diabetic Retinopathy Progression

VD has been described as a potential OCTA biomarker for DR progression in patients without DME. Xie N et al. [26] investigated the association of macular VD and DR and found that it was significantly reduced in patients with DR and was negatively correlated with the degree of retinopathy. Similar findings have been described by other authors: Forte et al. and Dimitrova et al. [27,28] reported a decrease in VD in diabetic patients, even without clinical signs of DR when compared to the controls. Li et al. [29] analyzed the VD of 97 diabetic patients and 48 controls and found that VD decreased in the diabetic patient group. Furthermore, Sun Z et al. [9] described that lower VD in the DCP was significantly associated with DR progression, whereas lower VD at the SCP was associated with DME development. This VD decrease may be explained by several factors, such as sustained hyperglycemia, which damages the blood–retinal barrier and promotes microvascular endothelial cell damage, leading to the narrowing and occlusion of the capillary network. Additionally, the inflammatory response that promotes exudation around the tissues with the secondary compression of the vascular network further aggravates the capillary occlusion and the degeneration of the macular vascularization.

### 3.3. VD Changes during DME Treatment

The impact of VEGF inhibition on macular vascularization during DME treatment is a subject of controversy. Some studies have reported an improvement in macular non-perfusion areas following anti-VEGF treatment [30,31]; others have not reported any statistically significant change in macular VD [32,33,34]. On the other hand, Elnahry et al. [35,36] reported decreased VD following intravitreal injections. These conflicting results may be due to OCTA technology itself, which is still in development and has several known limitations, affecting the interpretation of its results.

It is important to highlight that only a single mean VD value for the whole SCP and DCP was evaluated [30,31,32,33,34,35,36]. We hypothesize that if VD is not measured by regions, minor changes in areas with higher VD (due to microaneurysms and dilated shunt vessels) could easily be overlooked. By analyzing VD by region, we observed a trend towards lower VD values in all quadrants at month six, although the only statistically significant decrease was in the nasal DCP (−2.9 *p* 0.001).

The lack of relation between RT reduction and the changes seen in VD after treatment suggest that the latter does not represent the turnover of MAs alone and may be explained by other vascular phenomena induced by intravitreal injection flow that have been previously described by several authors, such as vasoconstriction, the repositioning of previously displaced vessels (Figure 1), and a reduction in moving particles that can be con-fused with blood.

Toto et al. [37], using laser speckle flowgraphy, reported a decrease in blood flow after intravitreal ranibizumab administration, which was associated with CST and BCVA. Fukami et al. [38] evaluated the effects of intravitreal injections in eyes with macular edema secondary to branch retinal vein occlusion, reporting transient vasoconstriction in the retinal arteries and veins and also reduced retinal blood flow.

It is also worth considering the influence of scattering particles in motion (SSPiM) [39] over VD metrics. Defined as an unusual extravascular signal on OCTA associated with the presence of hyperreflective material at the border of fluid spaces, it has been associated with artifactually increased VD in the foveal and perifoveal regions of the DCP. Maltsev DS et al. [40] evaluated the impact of SSPiM on VD in eyes with DME, finding that it was associated with artifactually increased VD in the foveal and peri-foveal regions of the DCP. If anti-VEGF treatment reduces the SSPiM in cystic areas, it could result in an “artificial” reduction in VD values.

All these factors, in conjunction with MA turnover, may be involved in the changes seen in VD values after anti-VEGF treatment.

The idea of being able to directly quantify the degree of MA reduction through an automatically quantifiable value such as VD in OCTA and correlate it with the treatment response during our daily clinical practice in a faster and safer way than through routine FA procedures would be a helpful tool for decision making during DME treatment. Chronic persistent DME can account for almost 40% of eyes treated with anti-VEGF therapies, according to the post hoc analysis of Protocol T and I from DRCR.net [41,42]. The prompt identification of the risk factors that can predict treatment success or failure is mandatory for reducing costs and patient burden. Traditionally, non-responders in clinical trial settings have been defined in relation to their response to treatment (less than three lines of visual gain, CST over 350 μm, or a reduction of less than 20% after six anti-VEGF injections), with the vascular changes often being relegated to having a secondary role due to the difficulty in acquiring routine vascular network measurements [43]. However, the evaluation of MAs pre- and post-anti-EGF therapy may have clinical implications for decision making. Hanato M et al., using indocyanine green angiography (IA), found that non-responders to anti-VEGF therapy had a higher number of pre-treatment nasal macular MAs [44]. Additionally, Mori et al. found that the number of MAs in late-phase IA at baseline and at six months was significantly higher in patients who had a recurrence of DME after treatment [45]. In these scenarios, the combination of different laser treatment regimens with anti-VEGF therapy plays an important role for improving disease control in the long term.

### 3.4. The Combination of Laser and Anti-VEGF Therapy for DME Treatment

The combination of different laser treatment regimens and anti-VEGF therapy has been studied in different clinical studies. The main objectives are to reduce the burden of intravitreal injections and to achieve better control of the edema, with less recurrences and better visual gains. Conventional laser photocoagulation therapy has the risk of RPE atrophy, which may potentially induce central scotoma with progressive enlargement. Therefore, in recent years, the development of new laser treatment techniques such as the navigated laser system Navilas and subthreshold micropulse laser therapy (SMLT) have gained popularity due to the lower risk of tissue destruction. Navilas has an eye-tracking delivery system that achieves greater accuracy (theoretically 60–100 μm) compared to conventional laser therapy for DME. Kato et al. [46] reported that navigated photocoagulation was effective for the treatment of 25 eyes with refractory DME, with improvements in CST being observed over six months. The laser photocoagulation was delivered using IA in most of the cases (23 cases); however, OCTA was used in 1 case with good results. Hirano et al. [47] reported that eyes with perifoveal leaking MAs required a larger number of anti-VEGF injections and that the addition of focal laser photocoagulation could reduce the number of injections and increase the BCVA by reducing the fluid influx to the fovea. However, targeting perifoveal MAs comes with the risk of foveal scaring with permanent vision loss. This is a scenario wherein a precise focal laser with Navilas (assisted by OCTA) could offer good results with fewer safety concerns.

The use of SMLT in combination with anti-VEGF injections for the treatment of DME has been investigated in some clinical studies that have yielded contradictory results, making it challenging to determine the overall effectiveness of the combination treatment in reducing the number of anti-VEGF injections. Abouhussein et al. [48], in a prospective trial of 40 eyes randomized to receive aflibercept monotherapy or aflibercept plus SMLT, reported a reduction in the number of injections in the combination group. On the other hand, in a similar study, Koushan et al. [49] did not find a significant reduction in the number of injections in the combination group. In theory, SMLT selectively targets RPE; therefore, the direct influence over MAs and telangiectasic vessels of the inner retina seems unlikely. However, Vujosevic et al. [50] reported changes in OCTA in 35 eyes with DME following treatment with SMLT for over a six-month period, characterized by a reduction in the foveal avascular zone area at the level of the deep capillary plexus and a reduced number of MAs and cystic areas in the SCP and DCP. The authors argue that although MAs are not the target of the SMLT, there is an indirect influence of the RPE over the regulation of the blood retinal barrier by releasing homeostatic factors that regulate the transport of ions, nutrients, oxygen, and water between the retina and the choroid.

Given the contradictory results reported in the literature regarding VD in DR in DME, it seems that VD is an OCTA biomarker that is prone to being influenced by many external factors, such as the device used to obtain the metrics, the way macular vascularization is addressed (unique value, foveal and parafoveal, quadrants), and the presence of cystic and vitreoretinal interface changes such as epiretinal membranes or vitreomacular traction, which may distort the capillary plexuses. Therefore, VD may be useful in predicting the risk of DR progression and DME development, but when the latter is established, its evaluation during treatment becomes complicated due to the several factors involved.

### 3.5. Strengths and Limitations

The present study has some limitations, including the heterogeneity of the included eyes, the wide range of visual acuity at baseline, its limited sample size, and variations in the duration and degree of loss to follow-up. However, the calculated sample size of 55 with up to a 10% of loss was met (48). By contrast, this study’s strengths include its prospective design, the homogenous treatment protocol, and the fact that all eyes were treatment-naïve. Another strength is the sector-by-sector approach used to assess VD and RT. The advantage of this approach, which differs from most previous studies, is that it may be more sensitive in detecting small changes in macular vascularization.

## 4. Conclusions

Our study’s findings reinforce the concept that macular vascularization is disrupted asymmetrically by DME. In DME-affected areas, we observed a pattern where capillary dropout and cystic changes (resulting in low vessel density or VD values) often coexist with areas characterized by a higher density of MAs, tortuous vessels, and vessel displacement due to edema (resulting in high VD values). Despite exploring VD as a potential OCTA biomarker in clinical practice, we could not establish a significant correlation between the reduction in RT and changes in VD, even when distinguishing between responders and non-responders. Consequently, the trend of VD reduction observed after six months might not accurately represent MA turnover alone, which has been linked to better treatment response in certain areas. This suggests that other vascular factors, such as vessel repositioning and changes in vessel caliber, may contribute to these observed VD changes. As a result, VD alone cannot serve as an indirect measure of MA turnover or the degree of treatment response to anti-VEGF therapy. Therefore, it seems that VD is an OCTA biomarker with a superior role in predicting disease progression rather than assessing treatment response.

## Figures and Tables

**Table 1:** Baseline characteristics of the participants of this study.

Systemic Features	Measurement	Ocular Features	Measurement
Age, years	63.7 (10.5)	BCVA, letters	69.7 (11.2)
Female sex	10 (29.4)	Central subfield thickness, µm	376.8 (100.3)
Type II diabetes	32 (94.1)	Treatment: Aflibercept Ranibizumab	33 (68.8) 15 (31.3)
HbA1C	7.84 (2.08)	Phakic	39 (81.3)
DR severity: Mild NPDR Moderate NPDR Severe NPDR PDR	4 (8.3) 20 (41.7) 6 (12.5) 18 (37.5)	Cataract: None N0 N1 N2 N3 PSC	8 (16.7) 15 (31.3) 18 (37.5) 4 (8.3) 2 (4.2) 1 (2.1)

Values represent mean (standard deviation) for quantitative variables and n (percentage) for categorical variables. Percentages may not add to 100% because of rounding. BCVA: best-corrected visual acuity; DR: diabetic retinopathy; HbA1C: glycated hemoglobin; NPDR: non-proliferative diabetic retinopathy; PDR: proliferative diabetic retinopathy; PSC: posterior subcapsular cataract.

**Table 2:** Changes in vessel density between baseline and month 6 in the SCP and DCP by sector.

	Superficial Capillary Plexus				Deep Capillary Plexus			
Sector	M0	M6	Diff	*p* Value	M0	M6	Diff	*p* Value
	%				%			
Superior	46.6 (3.8)	45.8 (4.4)	−0.7	0.28	52.5 (5.9)	51.4 (5.6)	−1.1	0.22
Inferior	46.0 (5.0)	45.7 (4.7)	−0.2	0.77	50.4 (4.9)	50.0 (5.4)	−0.4	0.71
Nasal	43.8 (4.3)	43.2 (3.9)	−0.7	0.27	50.2 (4.8)	47.4 (5.3)	−2.9	**0.001**
Temporal	44.6 (4.6)	43.7 (3.6)	−0.9	0.16	47.0 (5.6)	47.0 (5.9)	0.0	0.99
Mean	45.2 (3.5)	44.6 (3.2)	−0.7	0.16	50.0 (3.3)	49.0 (3.9)	−1.1	0.055

Abbreviations: SCP, superficial capillary plexus; DCP, deep capillary plexus; Diff: difference (month 6–month 0); M: month.

**Table 3:** Changes in BCVA and retinal thickness in each sector after treatment.

	Baseline	6 Months	*p*-Value
BCVA, letters	71.5 (14)	66.5 (16)	0.12
Center	354.5 (82.5)	306 (86)	<0.0001
Superior	342 (69.5)	329 (57)	<0.0001
Inferior	372 (87)	336.5 (77)	<0.0001
Nasal	346.5 (58.5)	337 (59.5)	<0.0001
Temporal	358 (82.5)	323 (62)	<0.0001

BCVA: best-corrected visual acuity. Values represent median (interquartile range).

**Table 4:** Spearman’s rho correlation coefficient for the association between retinal thickness and vessel density in different sectors, plexuses, and time periods related to treatment administration.

Sector	Coefficient	SE	*p*-Value	95% CI
**Superficial capillary plexus**				
Superior	−1.11	1.32	0.40	−3.69 to 1.47
Inferior	1.08	1.70	0.53	−2.25 to 4.41
Nasal	−2.17	1.18	0.07	−4.48 to 0.14
Temporal	−1.42	1.24	0.26	−3.85 to 1.02
**Deep capillary plexus**				
Superior	−0.11	1.69	0.95	−3.43 to 3.21
Inferior	1.50	1.93	0.44	−2.28 to 5.27
Nasal	−2.65	1.67	0.11	−5.92 to 0.62
Temporal	0.73	1.89	0.70	−2.98 to 4.45

Statistically significant results after Bonferroni adjustment (0.05/24 = 0.002).

**Table 5:** Differences between vessel density changes between responders and non-responders after 6 months of antiangiogenic therapy in diabetic macular edema. The analyses were repeated by sector and in each vascular plexus separately.

Sector	Coefficient	SE	*p*-Value	95% CI
**Superficial capillary plexus**				
Superior	−1.11	1.32	0.40	−3.69 to 1.47
Inferior	1.08	1.70	0.53	−2.25 to 4.41
Nasal	−2.17	1.18	0.07	−4.48 to 0.14
Temporal	−1.42	1.24	0.26	−3.85 to 1.02
**Deep capillary plexus**				
Superior	−0.11	1.69	0.95	−3.43 to 3.21
Inferior	1.50	1.93	0.44	−2.28 to 5.27
Nasal	−2.65	1.67	0.11	−5.92 to 0.62
Temporal	0.73	1.89	0.70	−2.98 to 4.45

## Data Availability

All data generated or analyzed during this study are included in this article. Further enquiries can be directed to the corresponding author.
